# A qualitative study to identify community structures for management of severe malaria: a basis for introducing rectal artesunate in the under five years children in Nakonde District of Zambia

**DOI:** 10.1186/1471-2458-5-28

**Published:** 2005-03-25

**Authors:** Frederick AD Kaona, Mary Tuba

**Affiliations:** 1 Directorate and Department of Behavioural Sciences, Mwengu Social and Health Research Centre, 12 Kafupi Road, Plot Number 1410/130 Northrise, P O Box 73693, Ndola, Zambia

## Abstract

**Background:**

Malaria is a serious illness among children aged 5 years and below in Zambia, which carries with it many adverse effects including anemia and high parasites exposure that lead to infant and childhood mortality. Due to poor accessibility to modern health facilities, malaria is normally managed at home using indigenous and cosmopolitan medicines. In view of problems and implications associated with management of severe malaria at home, rectal artesunate is being proposed as a first aid drug to slow down multiplication of parasites in children before accessing appropriate treatment.

**Methods:**

A qualitative study using standardised in-depth and Focuss Group Discussions (FGDs) guides to collect information from four (4) villages in Nakonde district, was conducted between February and March 2004. The guides were administered on 29 key informants living in the community and those whose children were admitted in the health facility. Participants in the 12 FGDs came from the 4 participating villages. Participants and key informants were fathers, younger and older mothers including grandmothers and other influential people at household level. Others were traditional healers, headmen, village secretaries, tradtional birth attendants, church leaders and black smiths. FGDs and interview transcriptions were coded to identify common themes that were related to recognition, classification and naming of malaria illness, care-seeking behaviour and community treatment practices for severe malaria.

**Results:**

Parental prior knowledge of the disease was important as the majority of informants (23 out of 29) and participants (69 out of 97) mentioned four combined symptoms that were used to recognise severe malaria. The symptoms were excessive body hotness, convulsions, vomiting yellow things and bulging of the fontanelle. On the other hand, all informants mentioned two or more of symptoms associated with severe malaria. In all 12 FGDs, participants reported that treatment of severe malaria commenced with the family and moved into the community as the illness progressed. Although treatment of severe diarrheal effects, were common among the winamwanga, no rectal medicines to treat severe malaria were identified. Apart from the anti-malarial fansidar, which was mentioned by 23 in IDIs and 40 in FGDs, participants and informants also frequently mentioned indigenous medicines provided by healers and other respectable herbalists for repelling evil spirits, once a child had severe malaria. Mothers were the important arms for administration of ant-malarial drugs in the villages. Referrals began with healers to CHWs, where no CHWs existed healers directly referred sick children to the health facility.

**Conclusion:**

Our findings showed that there is a precedent for rectal application of traditional medicine for childhood illness. Therefore rectal artesunate may be a well-received intervention in Nakonde District, provided effective sensitisation, to mothers and CHWs is given which will strengthen the health care delivery system at community level.

## Background

The problem of malaria infection in the under five years children has been widely described [1-5]. It is responsible for up to three million deaths annually throughout the world, with Africa having more than 90% of this burden. Almost 3% of disability-adjusted life years are due to malaria mortality globally, of these 10% are in Africa [6]. One of the most significant complications of the disease is Malaria-induced anemia causing more deaths than any of the other conditions of this disease [7].

In Zambia malaria continues to be the leading cause of deaths and illnesses, responsible for 47% of all deaths [8]. The incidence of the disease, and related mortality, in children under the age of five years is even higher [9]. Contributing factors to high child case fatality are the sudden progression from the simple form of the disease to severe malaria and malaria-induced anaemia, exacerbated by a lack of timely or inappropriate treatment. Simoes and colleagues in a review of the management of severely ill children in sub Saharan Africa found that over half of these children die before reaching the health facility for specialised care [10].

Where new drugs such as rectal artesunate have been developed and used for the emergency management of acute malaria in patients who cannot take drugs by mouth and for whom parenteral treatment is indicated but not available, positive results have been recorded [11,12]. The treatment should be followed by effective oral or parenteral therapy for malaria as soon as possible. In providing emergency therapeutic coverage, the drug is intended to give significant therapeutic advantage in situations where, at present, treatment does not exist. There is a potential for this approach to save lives of patients [13].

A lack of knowledge at family level on transmission of malaria hampers prevention of the disease. In the same study Simoes and others have shown that malaria case fatality rates were related to environmental and socio-economic status rather than poor knowledge of the disease 'malaria' [10]. In rural Zambia prompt access to treatment is hampered by the distances between families and the health facilities [9,14]. As a consequence most malaria-related morbidity is treated at home and within the community by informal health providers and networks [15,16].

In a clinical trial the rectal administration of artesunate has resulted in adequate drug absorbtion in all subjects and 92% children had achieved a parasite density lower than 60% of baseline after 12 hours compared with 14% of those administerd parenteral Quinine. Results were shown to be highly significant [12]. Its use in the emergency management of acute malaria in patients who cannot take drugs by mouth but require urgent treatment has therefore been established. However, for a significant reduction in case fatality in children to be achieved, prompt administration is essential. In Zambian rural households this implies that the responsibility for administration of rectal artesunate must lie with the caretakers of young children or those providing health care services at village level. The treatment should then be followed by effective oral therapy for malaria at a recognized health facility as soon as possible.

In preparation for the introduction of rectally administered artesunate at population level it is necessary to gain an understanding in three areas; firstly whether caretakers can identify malaria in their children under 5 years and distinguish between severe manifestations of the disease; secondly whether they would administer a drug, per rectum, if this was made available to them and thirdly and of great importance, whether the caretakers will then proceed to take the sick child to the health facility for follow up oral treatment. This paper describes the finding of a study designed to address these gaps in knowledge to inform the design of a study introducing rectal administered artesunate into the community. More specifically the paper covers the following areas: how communities defined symptoms of severe malaria, treatment seeking behaviour; decision making processes at a household level and the current use of rectally administered preparations to treat malaria.

### Health services in Nakonde

Nakonde District is situated in Northern Province of Zambia, it was chosen to conduct this study here because it is usually hard to reach communities. Average distances to health facilities range between ten and sixty kilometers. The District Health Management Board [DHMB], at the helm, controls all government activities in health care provision including one District hospital for referred patients. Of twelve administrative *Wards *only seven boast a rural health centre servicing all 154 villages in the district.

Communication between families and the health centres is through the Neighbourhood Health Committees, established per 'zone' covering a catchment of 500 populations or 5 to 10 km radius. Community Health Worker (CHWs), trained Traditional Birth Attendants (TBAs) and Village Health Motivators (VHMs), who are trained by the District Health Management Board, also report directly to the Rural Health Centres. Community Health Workers cover between three and five villages in their catchment areas. The presence of local healers and untrained Traditional Birth Attendants (TBAs) providing indigenous health care has a direct effect on the health seeking behaviour of the families in the villages. The villages in Nakonde are spread over a vast area and are sparsely populated, making it difficult for the government health structures to be easily accessible and effective.

## Methods

The study was conducted in Nakonde District between February and March 2004. Data collected was qualitative in nature, primarily using key informant interviews. Focus group discussions provided a means of triangulation.

### Sample population

Primarily interviews were conducted with caretakers and influential relatives at household level. Caretakers comprised of fathers of the under five years children, younger and older mothers and grandmothers. In view of the important role of traditional healers and other community members, village headmen, village secretaries, Traditional Birth Attendants, church leaders and blacksmiths were interviewed. The Focus Group Discussions (FGDs) were conducted to provide more responses and accommodated the emergence of hidden issues from the respondents. Finally, interviews with health care providers working at the closest Rural Health Centre (at Mwenzo) and the next referral level, the District Hospital (at Nakonde) were conducted.

### Focus Group discussions and key informant interviews

To provide a means of triangulating the information, key informant interviews and focus group discussions were conducted. Key informants consisted of 12 community informants, 11 mothers of children admitted at either Mwenzo Rural Health Centre or Nakonde District Hospital for severe malaria and 6 health care providers working either at Mwenzo Rural Health Centre or Nakonde Rural Health Centre. A total of 12 Focus Group Discussions (FGD), distributed equally in the four villages, were conducted. They lasted for between forty minutes and one hour. Separate groups of younger mothers, fathers and older mothers were organised and lastly a discussion with opinion leaders (all genders) was conducted (Table 1).

**Table 1:** distribution of participants per village

	Young Mothers	Fathers of <5 years	Older Mothers	Opinion Leaders	Total
Village 1	1	1	0	1	3
Village 2	0	1	1	1	3
Village 3	1	1	1	0	3
Village 4	1	0	1	1	3
Total	3	3	3	3	12

The number of people participating in each FGD varied between six and ten. The major criteria for participating in the FGD was availability of an under five child in the care of the mother or caretaker. Only younger mothers were categorized according to their age groups (15–24 years). However, no age restrictions were imposed on older mothers, fathers of under five or opinion leaders who participated in the FGDs.

### Procedures

The scene was set by the research team who visited the study site prior to the interviews, explaining the purpose of the study. All those who participated in the study were identified by the research team. The village headmen mobilised participants for FGDs through the criteria provided by the investigators.

The interviews were conducted by local residents following a training session by the research team at Mwengu Social and Health Research Center. Despite the permission that was granted by the community leadership, individual participants were requested to consent participating in the study. This arrangement ensured that the quality of data was not compromised, as enough time was given to allow for respondents to gain confidence in the research assistants. The interviews with caretakers lasted about one hour and about two hours with traditional healers and other opinion leaders. At every stage, interviewees were encouraged to talk freely. Transcription was completed following each interview before moving on to the next one. The authors closely supervised both data collection and transcriptions of materials.

All the interviews were tape recorded, transcribed and translated from Chinamwanga to English. The authors listened to recordings and checked the accuracy of the transcripts.

During the course of the research, investigators took turns to witness an in-depth being conducted by the research assistants and, in some cases, assisted in manual recording of responses. At every interview, the presence of the investigator was critical. During focus group discussions the investigators actively participated as facilitators.

### Research tools

A field guide was developed for use by interviewers and piloted in a community with the same socio-economic characteristics as the study site. The guide provided an opportunity to explore various community treatment behaviours of severe malaria, questions were related to serious illnesses in the community; information about health care; local terminologies of malaria; symptoms suggestive of simple and severe malaria; causes and treatment of different symptoms for simple and severe malaria; access to health facilities; dispensing practices at community and health facility levels; health information and communication channels regarding malaria and finally knowledge and practices regarding administration of rectal medicines.

### Research assistants

Criteria for selection of research assistants were completion of 12 years of schooling, previous experience as a research assistant, ability to speak the local language, Chinamwanga, fluently, resident in Nakonde, mature and respected by the people in the community. The four research assistants selected were all males aged between 27–35 years. The research assistants had not had any medical training; this assisted in minimizing the likely interviewer bias.

### Training of research assistants

The research team conducted an eight-hour training session for ten days familiarizing research assistants with research tools and practicing interviews. They were also taught basic techniques of recording responses and probing, identifying informants and note-taking for easy transcription. The fundamentals of organizing and conducting focus groups discussions were also included in the training. Investigators demonstrated in-depth interviewing.

### Analysis

The authors read and re-read the transcripts and developed codes to identify important themes. All transcriptions were typed in MS Word and converted into an Atlas.ti readable text file. Each interview and focus group discussion was entered as a single file. There were 41 single files for analysis. The files were then coded and independently prepared matrices for interpretation of substantive themes were developed. The purpose of the texture analyses was to interpret the data and findings by interrelating codes and data, in order to create common concepts.

To achieve this, data was segmented into variables, coded and then frequencies of codes were electronically assigned. Focus group discussion and in-depth interview transcripts were coded to identify common themes related to recognition, classification and naming of malaria illness, care seeking behaviour and traditional treatment practices for severe malaria. Codes were used to find specific occurrences of common responses in the data, which could not be searched by simply applying text based search techniques. They were also used to classify different levels of summaries that fell under different variables, for purposes of comparison between FGDs and in-depth interviews, as well as between categories of informants and participants. The process was followed to ensure a systematic basis for frequencies of the views expressed by participants during discussions and interviews.

Codes were standardized in all files according to variable under analysis, in order to ensure inter-code reliability. Assessment and validity of information was made by re-reading the transcripts.

## Results

### General description of malaria

The results are presented in themes previously selected to meet the objectives of the study; identification of malaria (all types), causes of malaria, use of rectally administerd medicines, health seeking behaviour and decision-making processes. Information from twenty-nine (29) interviews, the point at which no new information was forthcoming, and 12 focus group discussions are presented.

### Identification of malaria

Malaria was recognized as being a common problem in the area. At least 24 out of 29 key informants and 78 among the 97 FGD participants continuously mentioned this. An in-depth interview with a grandmother caring for under five child reported this:

*There is this disease number one, malaria. The children have finished *[meaning many children have died], *when diarrhea starts, at the same time with vomiting, you haven't arrived in time and the child.. we have buried many children, and the child is dead. Eee, this is the disease, which has defeated us very, very much*.

In one of the focus group discussions with opinion leaders, an old man aged over 60 years expressing himself on the prevalence of malaria in the district said: *The biggest problem, which we face in this area, especially for young children, is malaria *[said it in English], *inzekema is common here*.

The Winamwanga at times considered fever as an illness on its own in the under five children. They used the term '*Inzekema*' to describe the syndrome of fever, chills, body pains and headache. The term fever was used synonymously with the English word malaria. The categories of variables varied as shown in table 2 by the variable criteria for 'fever' [malaria, simple and severe].

**Table 2:** Types of malaria and terms used in the local language

Indigenous word	English word
1. *Inzekema*	Malaria
*2. Impepo*	Simple malaria
*3. Ichinzekema*	Severe malaria

Two types of malaria were recognized, simple and severe, the latter being associated with convulsions. The Winamwanga use 'Umuwili ukulungua' as a general term to describe the symptom of fever. 'Impepo' which is translated as cold, includes body hotness, shivering, headache and general body pains. This is considered as less serious, as the 46-year-old traditional healer put it:

*There are two types of malaria. There is one where a child is shivering and the body is hot, that is one type of malaria, we call it impepo*.

The two symptoms associated with an episode of simple malaria, that were commonly mentioned by both informants and participants included shivering and body hotness.

The local term '*sinsa*' [convulsion], was one of the key symptoms used by FGD participants to describe the progression of an illness, *'it is big malaria' *they could say. Making a statement on severe malaria, the 46 year old traditional healer continued and said:

*The other type of malaria comes with convulsions [sinsa], this is the other type. There are 2 different types of convulsions [sinsa]*.

Describing severe malaria a 40 year old key informant said:

*Vomiting *[ukuluka] *yellow things, when he starts vomiting yellow things, shivering too much *[ukuzakaza], *coldness/hotness *[impepo], *this is how we know that this is big malaria*.

In focus group discussions 78 out of 97 participants recognized that vomiting yellow things, convulsions, bulging of the fontanelle and excessive body hotness were some of the symptoms associated with severe malaria. These participants included those who had at one time taken their children to either Nakonde district hospital or Mwenzo rural health Centre, Convulsions were associated with severe malaria. At this point the reference to fever changes and they call '*E malelya mukulu'*, meaning (big malaria). A 54-year-old opinion leader in one of the FGDs said:

*we see when a child starts to say things we cannot understand; in the end you see the child prostate/stretching *[wanyuluka, Informant stretches his hands upwards showing how the child stretches].

Among the Winamwanga, vomiting yellow things was a clear sign that the malaria was coming out and that the child was at least *breathing *[meaning was relieved]. During the FGDs, it was common for participants to interpret vomiting yellow things as a sign of relief and beginning of healing of the illness. One participant in the FGD with younger mothers reported:

That is when he is breathing; it means the illness is coming out

A mother aged 39 years old, whose under five child was admitted at Nakonde hospital, reported that though most children with severe malaria vomited, not all vomiting was malaria. To show how vomiting due to severe malaria could be identified, she went on to say:

*They look yellow and they are slippery, if it is severe malaria *[Inzekema]. *But if it is another disease, because illnesses come different, he/she may just be vomiting *[ukuluka] *yellow things only and we say maybe it is severe malaria [Inzekema]*. [Meaning, they are unsure of the diagnosis]

Caretakers were capable of identifying different causes of some symptoms, which included diarrhea [*Ukupolomya*]. A mother whose child was admitted at the hospital stressed on '*diarrhea' *that was caused by witchcraft and said,'

*Cannot be cured until you find the African medicines, those one which when you cut tattoos, if there is no witch who is increasing it, but when he is there washing it *[meaning diluting the medicine], *it is not cured easily. Child will keep on crying, keep on crying. That is how it is*.

It was common to have mothers associating symptoms such as diarrhea with witchcraft, not with the malaria episodes.

When analyses were made on symptoms of convulsion and bulging fontanelle, caretakers knew differences between convulsions that occurred because of epilepsy and convulsions due to severe malaria attacks. They were able to associate a bulging fontanelle due malaria and that resulting from other illnesses. A father of the under five, who was 52 years old, said,

*' there are two. There is that one suffered by young children... an illness, which they say *[akatuwi] *sinking of fontanel, it causes the fontanel not closing quickly, this one is *[ichapamutwe]. *Now those... those illnesses are which differentiate them from malaria. And that one...that*... [akatuwi], *when you use a malaria drug, you find a child still sick. It closes without working. We... oh, this person needs African medicines which the people have been using long ago for cure. That is when we go to those who know the medicines*.

Reported malaria infection ranged from 50 to 80% in all villages. The prevalent conditions were convulsions, vomiting, high fever and loss of appetite.

### Rectal medicines

Rectally administered medications are already being used in this community.

During an FGD with older women, one participant described how the medicine for Ilonda was administered, she reported:

*Yes, turning the child upside down Sikaona *[referring to the interviewer], *sprinkling him/her just there where the dirt comes out *[anus], *that is where I sprinkle... there since it is Ilonda*.

It was common for most informants and participants, to explain in detail how medicines given through the rectum were administered. In an interview with a male key informant, regarding administration of medicine for Ilonda, he said:

*We just apply it on the sores on the anus, bath in medicine in the water and put the powder inside the anus, and then some you drink it. [*Meaning medicine is applied on the rectum]. *But...but some they buy those things from Tanzania, which they put medicine and inject people *(those things, referring to syringes).

When asked whether they would be willing to let their children have the drug inserted into the rectum, almost all the participants said it was alright as long as it made their children reach a health facility where they would continue seeking further treatment. As one member of the FGD said, '*me... I would not care even if the medicine was inserted in the ears or nose, for as long as the child survived that is alright*

### Decision making

The results from this study showed that the decision to use a facility when a child had severe malaria lay with the mother. Most informants and participants stressed on this point. During a Focus Group Discussion with opinion leaders, when asked who takes the decision for the child to go to the health facility for treatment, all participants shouted *: We mama *[the mother].

In another FGD with older mothers, a participant aged 25 years, with seven years of schooling and having three children reported;

*Just myself the brains come to decide that here it is not looking well. Me... when I tell the father *[Father here meaning husband], *you father this child is sick, aah.... It is just malaria you are just troubling let us just buy 'to' panadol that's how sometimes he will go and buy panadol, but we who are taking care of a child *[meaning one sleeping closer to the child]. *I am the one who is knowing how the child is becoming, so when I see that the child has not slept, I tell him my dear this child has not slept its better we take him/her where...? To the hospital*.

During discussions with opinion leaders, the responsibility for the mother to take the first decision was strongly stressed, as was evident in the following discussion:

Inf.6: *It's the mother of the child*. Inf.3, *5 *and 7 *almost at the same time: Its the mother *Inf.4: *The very mother of a child, is she not the very one who can tell the father? That lets take the child to the hospital*. Inf.5: *She has the right *Inf.4: *Because she is the first one to know the disease, so it means she has the right to decide*.

According to this discussion, a father was only consulted in order to provide financial and material support, which enabled a mother to use services from specialised health facilities or service.

### Health seeking behaviour

The majority of the participants believed that once a child is sick, unless treated, it was inevitable that he/she would die. Households started using structures and medicines that they were familiar with, before they could start looking for help outside what they knew. The chart below demonstrates caretakers' movements.

Indigenous Healer  CHWs  Admitting Health Centre

During an in-depth interview with a mother admitted at the hospital, she reported:

*It is just like when you meet the disease... some days you can waste even three days. Now if you have not met the illness, some days they fail like that they come and change and say go and buy this such you change again it is just like when you meet the illness like when it meets *[referring to drugs bought and administered at home] *with the illness it can even take two days *[says it in English], *and the child is better*.

While attempting to treat symptoms associated with severe malaria, local individuals, such as vendors and other people who knew some indigenous medicines were consulted. During an FGD, one informant aged 34 years stressed on this point when she reported:

*and those which you find here *[respondent touching on her top of the head showing where it sinks] *it is getting in side, even those they treat in the village, some people even *here [referring to the group], *they treat it*.

The informants emphasizing treatment-seeking patterns reported thus:

*We get ourmedicine from wa sing'anga, here *(Pointing at a healer). *If things do not work, then we go to the hospital where doctors try*. (FGDs).

One informant reported: *we just use traditional medicines here. If we all fail, then we send them to the hospital. [*Q: Do you have someone in this village that gives drugs from the hospital?] *A: Yes, we have one, Mr. Sikaona, if he has medicine, that is why I am saying, when we fail we send to Mr. Sikaonga [referring to a CHW], then he knows what to do next*.

Normally, a healer would take between two and four days before a referral was made to a community health worker. It was common for both informants and participants to report this number of days. One key informant in the village said:

*It takes 2 days...3 days to 4 days and when you give a child treatment, you now see a child starts playing, now you know he/she is okay*.

In an interview with mothers whose children were admitted in hospital, it was common for them to mention this pattern. One informant aged 37 years old reported:

*We take him/her to the village doctor [referring to the CHW]... the reason why it takes days because we first go to the village doctor, that is why it takes days because he gives us a dose and then we go back to him, or go to the hospital, when there is no change... If it is Fansidar, we wait for four days, if she/he has not changed, we go back to the village doctor and the village doctor says, to go to the hospital. Then we lift the child to the hospital, that's all*.

## Discussion

The study showed that severe malaria was well described by the Nakonde residents and was consistent across all the age groups of participants and key informants. They were able to correctly define and differentiate simple from severe malaria (simple malaria known as 'inzekema intichi' and severe malaria 'ichinzekema).' Both participants and informants expressed high level of knowledge of symptoms of severe malaria 'ichinzekema', which was directly reflected in their definition of 'big' malaria and were able to report symptoms in pairs or in groups, which might appear one at a time, accompanied with high temperature. The studies among the Tumbuka of Lundazi in the Eastern part of Zambia [17,18] presented similar findings and were consistent with studies conducted in other African Countries [19-21]. There was however, in this study, a confusion with the bulging of the fontanelle, which people regarded as a condition for severe malaria, while the medical field associates bulging of fontanelle with meningitis.

The study showed that caretakers recognise fever as being associated with malaria, they can determine severe and simple malaria and can differentiate between convulsions due to malaria and those due to epilepsy. In respect of rectal administration of drugs, it was found that local practices included rectal administration of local medications already exist. To determine whether caretakers then proceed to take the sick child to the health facility for follow up oral treatment, several factors were apparent. Firstly it is the mothers who would make this decision, secondly that health seeking behaviour follows a pattern of using the local traditional healers/CHWs and then, if no success is found then taking the child to the health facility.

Mothers and other caretakers in Nakonde could also identify severe malaria with many other complications and distinguish between convulsions due to other conditions. For example, only convulsions that were accompanied by high fever were regarded as 'big malaria' or severe malaria. Tchinda and others [22] in their study showed that anaemia, hypoglycemia and jaundice were recognized by caretakers as complications of malaria, this was not identified in the Nakonde study [23-25]. These parameters are critical foundations for an intervention strategy into home-based management of malaria.

While the majority of participants and informants perceived malaria to be an inevitable part of life in Nakonde others believed the causes for symptoms included evil spirits. This has implications for the introduction of home-based management of malaria since the casting out of evil spirits will delay seeking of biomedical treatment.

### Rectal medicines

In this study, investigations were made on whether there were any local medicines that were administered rectally to treat acute illness. During the in-depth interviews, it was shown that, although rectally administered medicines were not used for treatment of severe malaria in the Nakonde community; it was common for rectal medicines to be used for treating sores/wounds resulting from episode/s of severe diarrhea. The rectal medicines were (Inkula), local herbs and some backs of trees, like (Umulombwa and Umulala ntemelale)]. Inkula was widely used in the Northern and most of the Eastern part of Zambia. Inkula is sometimes called cassia in English.

Treating 'Ilonda', sometimes a child was made to sit in cold or warm water in which the medicine had been soaking. It was believed medicines soaked in water would go in the wound and treat it. The other interesting finding from this study was that healers were able to administer rectal medicines using syringes that were bought from chemist shops in Tanzania. Medicines that were in powdered form could be injected in the child's rectum. Healers, who could not afford syringes, used local materials such as straws to inject the rectal medicines into the children's rectum. With this background, introducing rectal artesunate in the Nakonde community might not face any resistance, as people were already administering some of their medicines in the same way as the modern rectal artesunate.

We believe that the most important element in the intervention would be to provide accurate and correct information to caretakers. Other studies [26,27], had recommended that there was need for effective system of providing information, in order to influence treatment seeking behaviour for severe malaria.

CHWs have been identified as the first port of call, along with the indigenous healers, once home treatment had been given. However the lack of essential drugs available to these workers contributes to delay in providing appropriate treatment. This study suggests that the provision of artesunate rectal suppositories to these health workers for the initial management of malaria patients who were not able to take medication by mouth would also reduce case fatality.

### Women decision-making

In this study, results revealed that a woman had a right to decide when to take a sick child to the health facility. According to these findings, a father was only consulted in order to provide financial and material support, which enabled a mother to use services from specialised health facilities. This finding is consistent with a study by Kaona et al [4], which indicated that health care in homes in rural Zambia and rural Africa as a whole, was predominantly the responsibility of women.

Young women in particular, felt very strongly that a severely sick child should never be kept at home or would certainly die. They appreciated the fact that early treatment would help the family avoid expending the family's' meager resources. Moreover, participants and informants categorically stated that children affected by severe malaria should be quickly referred to the health centres. Residents were deeply concerned that even the health facilities run without the qualified staff. If emergency treatment has been given prior to arrival at the health facility then unqualified staff can provide the oral treatment required to complete the cure.

### Community treatment seeking behaviour

The people of Nakonde commonly manage malaria episodes at household level initially. This is consistent with similar studies in other parts of Africa [16,26,25]. Breman for example showed that over 80% of malaria cases in the under five children, were managed at home [28]. Home treatment of malaria, has both advantages and disadvantages, one major disadvantage is that it may delay appropriate treatment during the acute stage of the disease.

It was established in this study, that the family was able to use various methods including sponging which was most common. Applications of these medicines varied though most of the respondents mentioned preparations through mixing with the child's porridge and drinks. Situations like this, made mothers and other caretakers to rely heavily on the indigenous medicines from healers, which were not anti-malarial drugs. There is no doubt, therefore, that caretakers can be trusted to identify when they would need to give rectal artesunate. However, there will be need for some extra sensitisation for mothers.

The study by Savingry and colleagues [29], found that the traditional healers attended over 28% of all deaths that had occurred. This is true for most of the societies in Eastern and Southern part of Africa. The use of indigenous plants in the treatment of malaria in Africa was reported by Waako, Fob and Smith [30]. They demonstrated that communities in Uganda could use plants such as Aspilia africana and Momordica foetida, for malaria treatment. The Winamwanga people revealed an extensive knowledge of both modern and traditional medicines for treating malaria. The Nakonde people used various types of medicines, for instance, there were medicines that could be used to repel evil spirits as perceived cause of convulsions, however, the main local remedies for treating malaria were, 'namayoka', referred to as cinchona in English, which was taken orally by the children. They use medicines which included '*nankololwe, utunvumbe, imbozyo, isyoyo, inkowamatete *and *umupene wambuzi*, as repellants for evil spirits. Sensitization messages must therefore address the need for the rectal artesunate to be administerd before evil spirits are expelled.

### Limitations

1. Lack of knowledge of the local language by the PI, could have made the research assistants fail to stress important issues during interviews. However, the Co-PI who comes from the area, managed to complement this.

2. The study recruited younger men as research assistants, to interview elderly men and women. This could have affected the responses from some of the participants.

3. The sampled individuals were purposively selected; these have led the study to generalize the findings.

4. The nature of the qualitative study, does not allow a researcher to measure the significance of the occurrence of an event.

## Conclusion

Our findings showed that there is a precedent for rectal application of traditional medicine for childhood illness. Therefore rectal artesunate may be a well-received intervention in Nakonde District, provided effective sensitisation, to mothers and CHWs is given which will strengthen the health care delivery system at community level.

## Competing interests

The author(s) delclare that they have no competing interests.

## Authors' contributions

FADK the Principal author of this paper, assisted in the design of the project to be adapted to the country's situation. He was instrumental in data collection, supervision during data collection, analysis and the production of this manuscript. MT was PI to the project. She contributed to the design of the project, supervised all field activities including data collection, transcription and editing of typed manuscripts. She was instrumental in data analysis. She has worked closely with the Principal author in the production of the manuscript. Authors have read and approved the final manuscript.

**Table 3:** Local terminologies for describing symptoms of severe malaria and their interpretations

Local terminology	English word
1. *Sinsa*	Convulsions
2. *Ukuzanzauchila/usawawiya*	Confusion/mental disorder
3. *Wakapanga ama picture ngati akulola a film*	Hallucination
4. *Akakalila*	Prostration
5. *Insingo ikakalila*	Stiff neck
6. *Ukusanta*	Fitting
7. *Ukukana ukulya*	Loss of appetite
8. *Ukuchuluchila*	Twitching
9. *Ukutwinka*	Sweating
10. *Ukupolomia*	Diarrhea
11. *Icapamutwe*	Fontanelle
12. *Ukuluka inyonga*	Vomiting yellow stuff
13. *Ukuzakaza*	Shivering
14. *Ukulungula nkaninye*	Very hot body
15. *Ukunyonga*	Stomach pains

## Pre-publication history

The pre-publication history for this paper can be accessed here:


